# Identification of a Strong Anthocyanin Activator, *VbMYBA*, From Berries of *Vaccinium bracteatum* Thunb.

**DOI:** 10.3389/fpls.2021.697212

**Published:** 2021-12-06

**Authors:** Ya-Ling Zhang, Kui Lin-Wang, Nick W. Albert, Caitlin Elborough, Richard V. Espley, Christelle M. Andre, Zhi-Zhen Fang

**Affiliations:** ^1^Fruit Research Institute, Fujian Academy of Agricultural Sciences, Fuzhou, China; ^2^The New Zealand Institute for Plant and Food Research Limited, Mt Albert Research Centre, Auckland, New Zealand

**Keywords:** *Vaccinium Bracteatum* Thunb., anthocyanin biosynthesis, transcriptome, VbMYBA, R2R3 MYB

## Abstract

Wufanshu (*Vaccinium bracteatum* Thunb.), which is a wild member of the genus *Vaccinium*, accumulates high concentration of anthocyanin in its berries. In this study, the accumulated anthocyanins and their derivatives in Wufanshu berries were identified through UHPLC–MS/MS analysis. Candidate anthocyanin biosynthetic genes were identified from the transcriptome of Wufanshu berries. qRT-PCR analyses showed that the expression of anthocyanin structural genes correlated with anthocyanin accumulation in berries. The R2R3-MYB, *VbMYBA*, which is a homolog of anthocyanin promoting R2R3-MYBs from other *Vaccinium* species, was also identified. Transient expression of *VbMYBA* in *Nicotiana tabacum* leaves confirmed its role as an anthocyanin regulator, and produced a higher anthocyanin concentration when compared with blueberry *VcMYBA* expression. Dual-luciferase assays further showed that VbMYBA can activate the *DFR* and *UFGT* promoters from other *Vaccinium* species. VbMYBA has an additional 23 aa at the N terminus compared with blueberry VcMYBA, but this was shown not to affect the ability to regulate anthocyanins. Taken together, our results provide important information on the molecular mechanisms responsible for the high anthocyanin content in Wufanshu berries.

## Introduction

Wufanshu (*Vaccinium bracteatum* Thunb.) is a wild blueberry species belonging to the genus *Vaccinium*. It is an evergreen shrub or small tree widely distributed in hilly areas of China (Wang et al., 2013; Fan et al., 2019). In China, it is a traditional herbal medicine used to treat many diseases, such as inflammation, diarrhea, and skin eruptions (Wang et al., 2004; Fan et al., 2019; Zheng et al., 2019). Leaf juice of Wufanshu was used to stain and cook well-known local traditional food named Wu Fan in China (Zhang et al., 2014). Pigments derived from leaves of Wufanshu were also widely used to dye hair and clothing (Xu et al., 2017; Fan et al., 2020). Wufanshu leaves are rich in health-promoting compounds such as polysaccharides and flavonoids (Wang et al., 2013; Zhang et al., 2014; Fan et al., 2018). It has been reported that its extracts have health beneficial activities, such as tyrosinase inhibiting, anti-inflammatory, anti-fatigue, anti-diabetic and anti-cancer (Wang et al., 2010, 2013; Landa et al., 2014; Oh et al., 2018a,b; Fan et al., 2019). More particularly, the presence of secondary plant metabolites, such as anthocyanins (Lee et al., 2014), in Wufanshu berry extracts has been associated with its health-promoting activities such as anti-proliferative, anti-inflammatory and antidepressant-like activity (Landa et al., 2014; Oh et al., 2018a). The amount of anthocyanins in fruits of *Vaccinium* species varies between species and cultivars (Lohachoompol et al., 2008; Stevenson and Scalzo, 2012; Li et al., 2017). Cho et al. (2012) showed that the anthocyanin concentration in berries of *V. bracteatum* Thunb. was higher than that of *V*. *corymbosum*, but the basis for this is unknown.

Anthocyanins belong to the flavonoid class of polyphenolics and are produced by a complex biosynthetic pathway. This starts with the condensation of three molecules of malonyl CoA and one molecule of 4-coumaroyl CoA by phenylalanine ammonia-lyase (PAL), cinnamate-4-hydroxylase (C4H), 4-coumaroyl:CoA-ligase (4CL), chalcone synthase (CHS). The naringenin chalcone formed from this is then converted to naringenin flavanone by chalcone isomerase (CHI), and then hydroxylated by flavanone-3-hydroxylase (F3H), to form dihydrokaempferol. At this stage the pathway can branch to form different anthocyanins, such as cyanidin or delphinidin. Further hydroxyl groups are added at this stage by flavonoid3′-hydroxylase (F3′H) or flavonoid 3′, 5′-hydroxylase (F3′5′H) before conversion by dihydroflavonol-4-reductase (DFR). The formation of anthocyanidins (aglycones) is controlled by anthocyanidin synthase (ANS), and these are glycosylated by UDPglucose:flavonoid-3-*O*-glucosyltransferase (UFGT) to form anthocyanins (Holton and Cornish, 1995). Additional chemical modifications of the basic anthocyanin molecules with additional sugars, methyl and acyl groups can generate a large variety of anthocyanins that differ in color and chemical properties. Many of these biosynthetic steps are shared with proanthocyanidin (condensed tannin) biosynthesis, but the activity of UFGT is necessary for anthocyanin production. Thus, it is a key point of regulation in fruit species that have complex flavonoid profiles, such as grape and *Vaccinium* spp. (Kobayashi et al., 2002; Günther et al., 2020).

In *Vaccinium* species, such as blueberry, the most common anthocyanins are aglycones of cyanidin (cyanidin 3-galactoside, cyanidin 3-arabinoside and cyanidin 3-glucoside), peonidin (peonidin 3-glucoside, peonidin 3-galactoside and peonidin 3-arabinoside), delphinidin (delphinidin 3-galactoside, delphinidin 3-glucoside and delphinidin 3-arabinoside), malvidin (malvidin 3-arabinoside), and petunidin (petunidin 3-galactoside, petunidin 3-arabinoside and petunidin 3-glucoside) (Lohachoompol et al., 2008). The network that regulates anthocyanin biosynthesis in plants has been studied extensively (Jaakola, 2013). While all three components are necessary for anthocyanin regulation, the central component of the network that regulates anthocyanin biosynthesis is the MYB-bHLH-WD40 (MBW) complex (Nesi et al., 2001; Gonzalez et al., 2008; Albert et al., 2014) and R2R3-MYBs have been shown to be the key regulators across a range of plant species (Espley et al., 2007; Hichri et al., 2011; Albert et al., 2014; Feng et al., 2015; Tuan et al., 2015; Jin et al., 2016; Butelli et al., 2017; Plunkett et al., 2018; Zhang et al., 2018). This is because the MYB protein is usually the limiting component of the MBW complex, and because the MYB can activate the expression of bHLH and WDR genes through a widely conserved hierarchy (Albert et al., 2014, 2021). In *Vaccinium* species, R2R3-MYBs have been reported to be involved in anthocyanin accumulation (Nguyen et al., 2017; Plunkett et al., 2018; Die et al., 2020) and down regulation of their expression was associated with a reduction or loss of anthocyanin accumulation in berries (Primetta et al., 2015; Yang et al., 2018; Die et al., 2020). The regulators of anthocyanin biosynthesis of Wufanshu have not yet been identified. Understanding the regulatory mechanisms in these the highly anthocyanic berries will increase our knowledge of anthocyanin accumulation in the *Vaccinium* genus.

In this study, we report on the identification of *VbMYBA*, which is closely related to the R2R3-MYB anthocyanin activator *VcMYBA* from blueberries (*V. corymbosum* and *V. virgatum*) (Plunkett et al., 2018) and show that its anthocyanin promoting activity is stronger than *VcMYBA*. Our results will help the development of new *Vaccinium* cultivars that can accumulate more anthocyanins in their berries.

## Materials and Methods

### Plant Materials

All berries at different stages were harvested from wild Wufanshu plants in Lianjiang, in the city of Fuzhou (Fujian Province, China). Green berries were harvested on June 19, 2016. Red and black berries were harvested on November 16, 2016 and divided into two groups according to color. We estimated that these berry stages approximately correspond with stages 5, 6, and 8, described by for blueberry (Zifkin et al., 2011). Berries sampled from three different plants were used as biological replicates. For each plant, berries with similar size and color were pooled together. After harvesting, all samples were transported to lab and immediately frozen in liquid nitrogen and kept at −80°C for further analysis.

### Total Anthocyanin Content Analysis in Wufanshu Berries

Approximately 1 g (FW) of whole berries was ground to fine powder in liquid nitrogen and extracted with 10 mL extraction solution (0.05% HCl in methanol) at 4°C for 24 h. Anthocyanin content was quantified using pH differential method as described previously (Fang et al., 2016).

### Anthocyanins Characterization of Wufanshu Berries

Extraction, identification and quantification of metabolites were carried out at Suzhou BioNovoGene (Suzhou, China). Approximately 0.2 g (FW) of the berry powder was transferred to a 2 mL centrifuge tube containing 1 mL of water: methanol: formic acid (66.5: 28.5: 5, v/v/v), incubate on ice for 30 min and centrifuged at 18,756 × *g* for 10 min. The tube containing the mixture was centrifuged at 12,000 rpm for 10 min. The supernatants were freeze-dried and the extracts were resuspended with 0.2 mL 5% formic acid. An Ultra-high performance chromatography system (UHPLC, Vanquish, Thermo Fisher Scientific, Waltham, MA, United States) with a Waters HSS T3 (2.1 × 100 mm, 1.8 μm) column was used. The parameters used were as follows: flow rate of 300 μL min^–1^, column oven temperature of 40°C, and sample size of 2 μL. The mobile phases were 0.1% formic acid in water (A) and 0.1% formic acid in acetonitrile (B). The gradient elution procedure was as follows: 0-4.0 min, 10% A; 4.0-12.0 min, 10–60% A; 12.0–18.0 min, 60% A constant; 18.0–18.1 min, 60-10% A; 18.1–26.0 min, 10% A constant. Mass spectrometry (MS) analysis was carried out on a Thermo Q Exactive instrument (Thermo Fisher Scientific, Waltham, MA, United States) equipped with an electrospray ion (ESI) source in positive mode. The capillary voltage was set to 3000 V and the capillary temperature was set to 320°C. The pressure of sheath gas and auxiliary gas were set at 40 and 10 arbitrary units, respectively. Metabolites were detected using full-scan/MS2 mode with a resolution of 70000. ESI spectra were acquired through the information-dependent acquisition mode in Xcalibur 4.1 software (Thermo Fisher Scientific, Waltham, MA, United States). The dynamic exclusion time was set to 6s. For MS1, full MS spectra between 200 and 1500 mass-to-charge ratio (m/z) were recorded. MS/MS scans were recorded between 200 and 2000 m/z. Data dependent acquisition survey scans were acquired in 100 ms and the 10 most abundant product ion scans were collected. Each metabolite was confirmed based on their exact molecular weights, then the fragment information obtained according to the MS/MS mode was matched in database built by BioNovogene to identify metabolites. Searches were performed using the following settings: precursor ion m/z tolerance: ±10ppm; MS/MS m/z tolerance: ±20ppm.

### RNA Extraction, cDNA Library Construction and Sequencing

For RNA-Seq, berries at green or black stage from three replicates were mixed and subjected to RNA extraction. Total RNA extraction, cDNA library construction, and sequencing were performed by staff at Beijing BioMarker Technologies (Beijing, China). RNA extraction was carried out using EZNA Plant RNA Kit (Omega Bio-tek) RNA purity was checked using the NanoPhotometer^®^ spectrophotometer (IMPLEN, CA, United States). RNA concentration was measured using Qubit^®^ RNA Assay Kit in Qubit 2.0 Fluorometer (Life Technologies, CA, United States). RNA integrity was assessed using the RNA Nano 6000 Assay Kit of the Agilent Bioanalyzer 2100 system (Agilent Technologies, CA, United States). cDNA library was generated using NEBNext^®^Ultra^TM^ RNA Library Prep Kit for Illumina^®^(NEB, United States) following manufacturer’s recommendation. Library quality was assessed using the Agilent Bioanalyzer 2100 system. Sequencing of the cDNA libraries were carried out on the Illumina HiSeq^TM^ X Ten sequencing platform. The RNA-seq reads have been deposited in the NCBI Short Read Archive and are accessible under PRJNA694726.

### *De novo* Assembly, UniGene Annotation and Expression Quantification of Unigenes

The raw reads were processed through in-house perl scripts and *de novo* assembled into unigenes with Trinity (Grabherr et al., 2011) as described previously (Zhang et al., 2018). Annotation of the assembled unigenes was performed using BLASTx (E-value<10–5) searches against eight public databases including the NCBI non-redundant protein database (Nr), Swiss-Prot protein database (Swiss-Prot), the Gene Ontology database (GO), the Clusters of Orthologous Groups database (COG), the euKaryotic Ortholog Groups of proteins database (KOG), the Kyoto Encyclopedia of Genes and Genomes (KEGG), the Pfam-protein family database (Pfam) and the evolutionary genealogy of genes: Non-supervised Orthologous Groups database (eggNOG). For calculation of gene expression level, clean RNA-Seq reads were mapped to the assembled unigenes using Bowtie (Langmead et al., 2009) and the expression levels of unigenes were calculated with fragments per kilobase per million mapped reads (FPKM) using RSEM (Li and Dewey, 2011).

### qRT-PCR (Real-Time Quantitative RT-PCR) Analysis

Total RNA was extracted from green, red and black Wufanshu berries using EZNA Plant RNA Kit (Omega Bio-tek). First strand cDNA was prepared from 500 ng total RNA HiScript III RT SuperMix for qPCR with gDNA wiper (Vazyme, Nanjing, China). qRT-PCR was performed using the Eppendorf Realplex^4^ real-time PCR system (Hamburg, Germany) in a total volume of 20 μL in each well containing 10 μL of 2 × ChamQ Universal SYBR qPCR Master Mix (Vazyme, Nanjing, China), 6 μL of cDNA (in 1:30 dilution), and 0.4 μL 10 μM primers. qRT-PCR conditions were 30 s at 95°C, followed by 40 cycles of 5 s at 95°C, 15 s at 60°C, followed by 60°C to 95°C melting curve detection. *Actin* gene (c87909.graph_c0) was used as the reference. The expression levels were calculated using the 2^–ΔΔCT^ method. Three biological and four technical replications were performed. Primers for qRT-PCR were listed in Supplementary Table 1. Linear regression analysis of FPKM and qPCR was performed using Minitab^®^ 18.

### Cloning of *VbMYBA* and Prediction of Amino Acid Sequence

cDNA of black Wufanshu berries was generated using a First-Strand cDNA synthesis kit (Fermentas, United States) and used as template for gene cloning. The cDNA sequence encoding VbMYBA was isolated using I-5^TM^ 2 × High-Fidelity Master Mix (MCLAB, San Francisco, CA) with primers (forward 5′-GGCAGCTTACATGAAAATTCTCC-3′ and reverse 5′-CAAACAAAGAAATGCTTGCCG-3′) designed according to unigene sequence. The generated PCR products were purified and subsequently cloned into the pEASY-Blunt Zero vector using pEASY-Blunt Zero Cloning Kit (TransGen, Beijing, China) and sequenced. The open-reading frame of the sequence was predicted using GenBank ORF finder^1^.

### Multiple Sequence Alignment and Phylogenetic Analysis

Multiple sequence alignment of R2R3 MYB amino acid sequences from Wufanshu, blueberry, kiwifruit and peach was performed by CLUSTALW^2^. Shading of the alignment results was performed using ESPript 3.0 (Robert and Gouet, 2014). Phylogenetic tree was constructed using the maximum likelihood method with 1000 bootstrap replicates by MEGA-X. R2R3 MYB amino acid sequences used for phylogenetic analysis include *Arabidopsis thaliana* AtMYB4 (At4G38620), AtMYB11 (AT3G62610), AtMYB12 (AT2G47460), AtMYB75 (AT1G56650), AtMYB90 (AT1G66390), AtMYB111 (AT5G49330), AtMYB113 (AT1G66370), AtMYB114 (AT1G66380), AtMYB123 (AT5G35550); *Actinidia chinensis* AcMYB10 (PSS35990), and AcMYB110 (AHY00342); *Fragaria* × *ananassa* FaMYB1 (AAK84064.1), FaMYB9 (JQ989281), FaMYB10 (ABX79947.1); *Gossypium hirsutum* GhMYB1 (AAA33067.1), GhMYB6 (AAN28286.1) GhMYB10 (ABR01222.1), and GhMYB36 (AF336284); *Lotus japonicus* LjMYB12 (AB334529); *Myrciaria cauliflora* McMYB (MH383068); *Malus* × *domestica* MdMYB1 (XP_028963316.1), MdMYB9 (DQ267900), MdMYB10 (EU518249), MdMYB11 (DQ074463); *Medicago truncatula* MtLAP1 (ACN79541.1), and MtMYB14 (XP_003594801.1); *Prunus cerasifera* PcMYB10.1 (KP772281) and PcMYB10.2 (KP772282); *Petunia hybrida* PhMYB27 (AHX24372), PhAN2 (AB982128), PhDEEP PURPLE (ADQ00393.1), PhPURPLE HAZE (ADQ00388.1) and PhMYB4 (ADX33331.1); *Prunus persica* PpMYB10.1 (XM_007216468), PpMYB10.2 (XM_007216161) and PpMYB18 (KT159234); *Prunus salicina* PsMYB10.1 (MK105923), PsMYB10.2 (MK340932), and PsMYB18 (MK284223); PtMYB14 (ACR83705.1); *Pyrus pyrifolia* PyMYB10 (GU253310); *Solanum lycopersicum* SlMYB12 (ACB46530.1); *Trifolium arvense* TaMYB14 (AFJ53053.1); *Trifolium repens* TrMYB4 AMB27079), TrMYB133 (AMB27081), and TrMYB134 (AMB27082); *Vaccinium ashei* VaMYB (QOQ50851.1), *Vaccinium corymbosum* VcMYBA (MH105054); *Vaccinium uliginosum* VuMYBC2; *Vitis vinifera* VvMYBA2 (BAD18978), VvMYBF1 (ACV81697), VvMYBC2-L1 (AFX64995.1), VvMYB-L2 (ACX50288.2), and VvMYBPA2 (ACK56131.1).

### Vector Construction

Full-length coding sequence of *VbMYBA* was isolated using 2 × Phanta Max Master Mix (Vazyme, Nanjing, China) with primer VbMYBAOEF and VbMYBAOER and inserted into pSAK277. A fragment (70–855 bp) of *VbMYBA* was amplified by primer VbMYB-NdelOEF and VbMYB-NdelOER. Fusion PCR was carried out to generate VcMYBA-Nadd (*VcMYBA* with 1–69 bp of *VbMYBA*) were constructed as following: the N-terminal of VbMYBA (1–69 bp) was amplified by primer VbMYBNF and VbMYBNR, and the *VcMYBA* contains overlap with N-terminal of *VbMYBA* (1–69 bp) was amplified by VcMYBF and VcMYBR. Then the two fragments were fused through PCR. Finally, the VcMYBA-Nadd was amplified by primer VbMYB-NaddOEF and VbMYB-NaddOER, and inserted into pSAK277. The insertion of PCR fragments into pSAK277 was conducted using ClonExpress Ultra One Step Cloning Kit (Vazyme, Nanjing, China). Primer sequences used for the vector construction are listed in Supplementary Table 2. The promoter of *VcDFR* was previously isolated (Plunkett et al., 2018) and the promoter of *VavUFGT* was isolated from *Vaccinium virgatum* using the primers VavpUFGT-F (CTCCACATTTTTAACCTGGTGCAC) and VavpUFGT-R (CATGGTTATATTTTTGGTGGT), and cloned into pGreenII 0800-LUC (Hellens et al., 2005).

### Transient Transformation in Tobacco Leaf and Quantification of Anthocyanins

Transient color assays were carried out in young leaves of *Nicotiana tabacum* seedlings grown in the greenhouse as described previously (Fang et al., 2021). All the constructs were transformed into *Agrobacterium tumefaciens strain GV3101*, and incubated at 28°C for 2 days. *Agrobacterium* cultures carrying constructs were resuspended in infiltration buffer containing 10 mM MgCl_2_ and 100 μM acetosyringone (pH = 5.7) and incubated at room temperature without shaking for 2 h before infiltration. Mixed bacterial cultures were injected into the abaxial side of young leaves. Many studies have suggested that basic helix-loop-helix (bHLH) transcription factors are indispensable partners of R2R3-MYBs (Espley et al., 2007; Gonzalez et al., 2008), in particular those belonging to the bHLH2 subgroup (PhAN1/AtTT8). Tobacco leaves express WDR and bHLH1 (PhJAF13/AtEGL3) orthologs, but require a bHLH2 (PhAn1/AtTT8) protein to fully regulate anthocyanin biosynthesis (Montefiori et al., 2015). Because the ability for the MYB to regulate the endogenous tobacco *bHLH2* gene is variable in transient assays, a *bHLH2* (*PsbHLH3*) gene was co-transformed. We failed to clone the anthocyanin-associated bHLH from Wufanshu, and so we chose the plum anthocyanin-activating PsbHLH3 for transient color assays in tobacco leaves (Fang et al., 2021). This is a close homolog of peach PpbHLH3, which showed stronger anthocyanin-promoting activity than the homologous apple MdbHLH3 when coinfiltrated with blueberry VcMYBA (Plunkett et al., 2018). Separate strains containing *VbMYBA* and *PsbHLH3* fused to the 35S promoter in the pSAK277 vector and empty pSAK277 vector were infiltrated or co-infiltrated into the abaxial leaf surface. Blueberry *VcMYBA* (Plunkett et al., 2018) co-infiltrated with *PsbHLH3* was used as positive control. Each infiltration was performed using three leaves from the same plants. Photographs were taken seven days after infiltration.

For quantification of anthocyanins, 10 mg of freeze-dried tobacco leaves from the infiltrated area was mixed in 1 mL of methanol: water: formic acid (80: 19.5: 0.5, v/v/v) and shaken for 4 h at room temperature. The tube containing the mixture was centrifuged at 10,000 × *g* for 15 min. The supernatants were filtered through a 0.45 μm PTFE syringe filter and submitted to high performance liquid chromatography (HPLC) analysis according to a method reported by Andre et al. (2012) with a few modifications. Briefly, quantification of the anthocyanins was performed using a Dionex Ultimate 3000 system (Sunnyvale, CA, United States) equipped with a diode array detector (DAD). A 5 μL aliquot was injected onto a Thermo C18 Acclaim PolarAdvantage II column (150 × 2.1 mm i.d.; 3 μm particle size) (Waltham, MA, United States). The mobile phases were (A) H_2_O with 5% formic acid and (B) MeCN with 0.1% formic acid. The flow rate was 0.35 mL min^–1^, and the column temperature was 35°C. The 28 min gradient was as follows: 0-5 min, 7% B constant; 5-10 min, 7-12% B; 10-20 min, 12-25% B; 20-21 min, 25-100% B; 21-24 min, 100% B constant; 24 min, 7% B; 24-28 min, 7% B re-equilibration time. Monitoring was set at 520 nm for quantification. Anthocyanins were identified by their spectral data and were quantified as cyanidin-3-glucoside using five-point calibration curves. A validation standard was injected after every 10th injection.

### Dual-Luciferase Assay

Dual-luciferase assays were conducted in *Nicotiana benthamiana* leaves as reported previously (Lin-Wang et al., 2010). All the promoter constructs were individually transformed into *Agrobacterium* strain GV3101 that contains the pSoup plasmid using the electroporation method. Agrobacteria cultivation and infiltration preparation were performed according to the same protocol as described above for the transient color assay.

## Results

### Accumulation of Anthocyanin in Berries of Wufanshu

**FIGURE 1:**
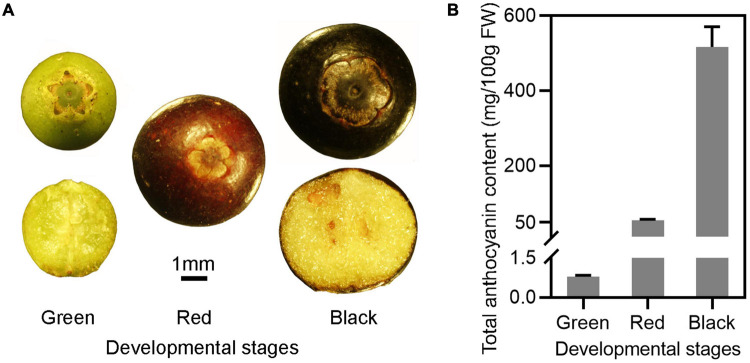
Anthocyanin accumulation in the fruits of Wufanshu. **(A)** Fruits of Wufanshu at different stages. **(B)** Anthocyanin content in fruits of Wufanshu at different ripening stages. Error bars represent standard error of three replicates. Error bars represent standard error of five replicates.

The skin of unripe Wufanshu berries was initially green, then turned red and finally black when fully ripen (Figure 1A). The total content in anthocyanins increased during the ripening of berries and reached 516.97 mg/100 g FW at the full ripe stage (Figure 1B). Individual anthocyanin compounds were characterized in ripe Wufanshu berries using UHPLC–MS/MS analysis. Delphinidin, cyanidin, malvidin, peonidin, petunidin, as well as pelargonidin derivatives could be identified based on their mass spectral data. Delphinidin 3-galactoside and 3-glucoside predominated the anthocyanin profile (Supplementary Table 3).

### RNA Sequencing and *de novo* Assembly of Wufanshu Berry Transcriptomes

To identify genes responsible for anthocyanin accumulation in the berries of Wufanshu and investigate the underlying molecular mechanisms, transcriptomes of berries at green and black stages were generated by RNA-Seq. A total of 13.49 Gb clean reads were generated (Supplementary Table 4) and *de novo*-assembled into 87,811 unigenes. The average length of unigenes was 672.45 nt. Over one-half of the unigenes (61.59%) were shorter than 500 bp, and only 18.59% (16,319) of the unigenes were longer than 1 kb (Supplementary Table 5).

All unigenes were annotated against eight public protein databases; NR, Swiss-Prot, Pfam, GO, COG, KOG, eggNOG4.5 and KEGG. In total, 49.37% (43,355) of the unigenes showed significant BLAST matches to known sequences in these databases (Supplementary Table 6). According to the KEGG enrichment analysis, 168 unigenes were assigned to phenylpropanoid biosynthesis pathway (ko00940), 48 unigenes were assigned to flavonoid biosynthesis pathway (ko00941) and fourteen genes were involved in the anthocyanin biosynthesis pathway (ko00942) (Supplementary Table 7). Twenty-seven of these genes, which were more abundant in transcript levels in black Wufanshu berries (Supplementary Table 8), were identified as candidate genes involved in anthocyanin biosynthesis. c81310.graph_c0 was annotated as leucoanthocyanidin dioxygenase according to Swissprot database and c84705.graph_c0 was annotated as UDP-glucose: flavonoid 3-*O*-glucosyltransferase according to Nr database (Supplementary Table 8). In addition, c72505.graph_c0 was predicted to encode a homolog of cyclamen CkmGST3, reported to be involved in anthocyanin accumulation (Kitamura et al., 2012).

### Anthocyanin Biosynthetic Genes Are Upregulated in the Berries of Wufanshu

**FIGURE 2:**
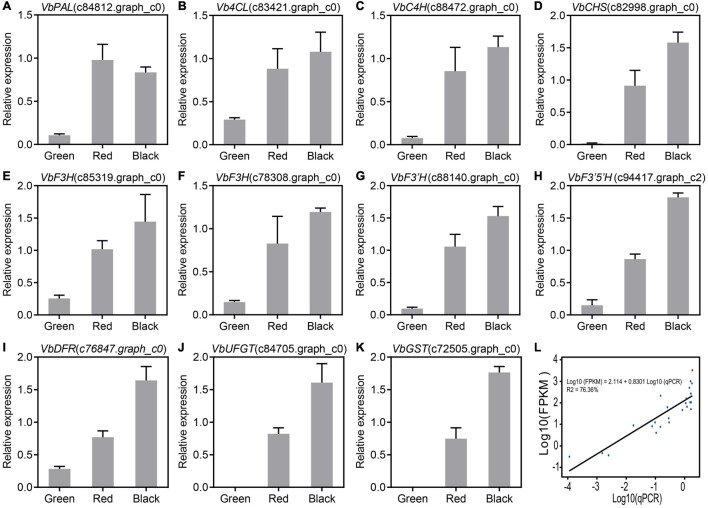
Expression of candidate genes involved in anthocyanin biosynthesis. **(A–K)** qRT-PCR analysis of candidate genes involved in anthocyanin biosynthesis. *Actin* was used as the reference gene. The error bars represent the standard error of three biological replicates. **(L)** Linear regression analysis of gene expression levels obtained from RNA-Seq and quantitative real-time PCR data.

Based on the RNA-Seq data, the expression levels of 11 anthocyanin biosynthetic genes with high FPKM values (maximum FPKM > 40) were analyzed by qRT-PCR. Our results indicated that the expression of *VbPAL* (*phenylalanine ammonia-lyase*, c84812.graph_c0), *Vb4CL* (*4-coumaroyl:CoA-ligase*, c83421.graph_c0), *VbC4H* (*cinnamate-4-hydroxylase*, c88472.graph_c0), *VbCHS* (*chalcone synthase*, c82998.graph_c0), *VbF3H* (*flavanone-3-hydroxylase*, c85319.graph_c0 and c78308.graph_c0), *VbF3′H* (*flavonoid3′-hydroxylase*, c88140.graph_c0), *VbF3′5′H* (c94417.graph_c2), *VbDFR* (*dihydroflavonol-4-reductase*, c76847.graph_c0), *VbUFGT* (*UDPglucose:flavonoid-3-O-glucosyltransferase*, c84705.graph_c0), and *VbGST* (*glutathione S-transferase*, c81305.graph_c0) were upregulated during ripening of Wufanshu berries (Figures 2A–K). Linear regression analysis showed that qRT-PCR value of the analyzed anthocyanin biosynthetic genes was significantly correlated with that FPKM value of them (Figure 2L).

### Identification of *VbMYBA* From Wufanshu Berries

**FIGURE 3:**
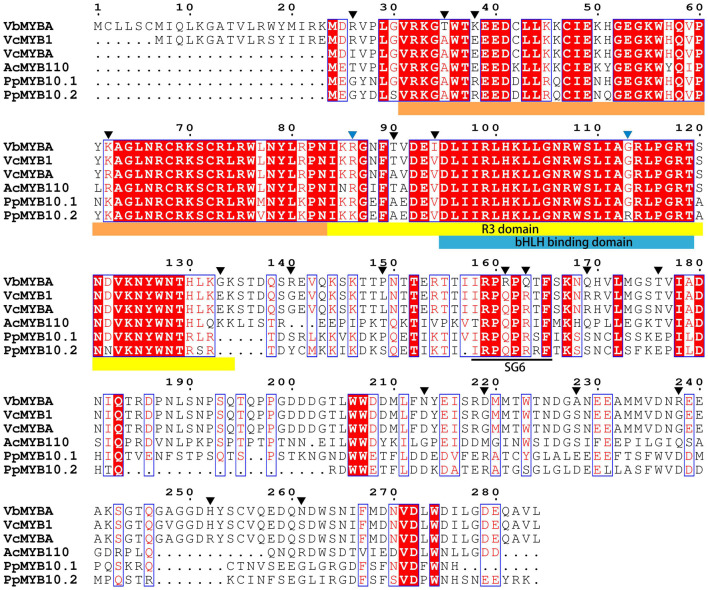
Sequence alignment of VbMYBA and anthocyanin MYB activators from blueberry and peach. R2, R3, and bHLH binding domain are highlighted in marigold yellow, yellow, and blue colors, conserved SG6 motif for anthocyanin-promoting MYBs are indicated in black line. Amino acids that are different between VbMYB and blueberry VcMYB were indicate with black triangles. Amino acids that were reported to affect the anthocyanin-promoting activity of MYB proteins (Zhou et al., 2018) are indicate with blue triangles.

**FIGURE 4:**
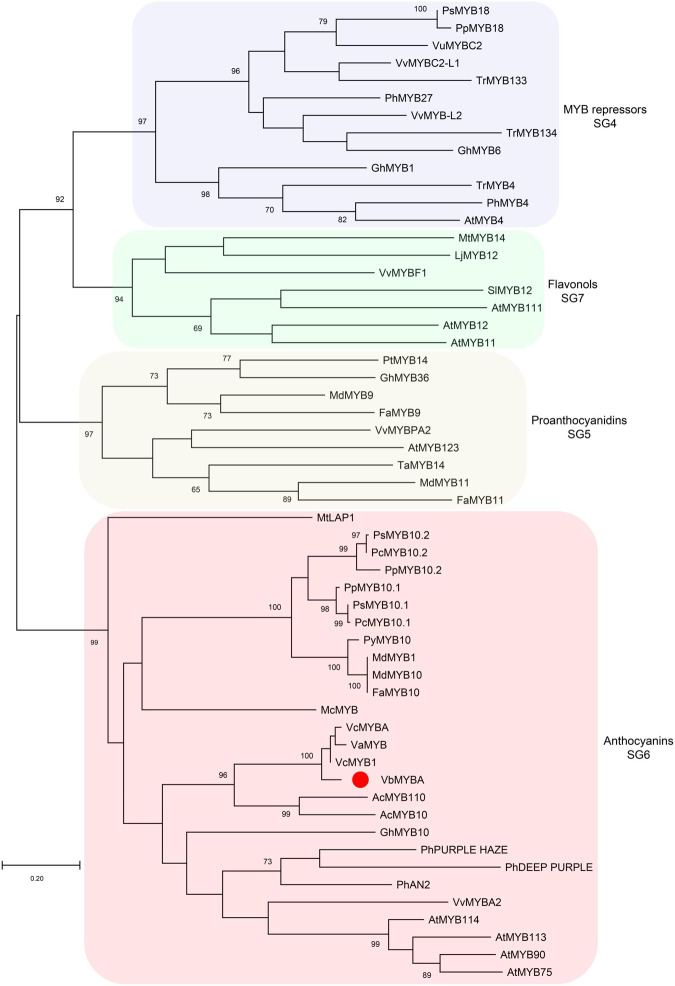
Phylogenetic analysis of VbMYBA and R2R3 MYBs from other plant species. Phylogenetic tree was constructed using maximum-likelihood method by MEGA-X software. VbMYB from Wufanshu fruits is indicated with red dot. Phylogenetic groups are highlighted with different color shades. Numbers indicate node support >65% from 1000 bootstrap replicates. The scale bar represents 0.2 substitutions per site.

To identify candidate anthocyanin MYB activators, amino acid sequence of VcMYBA (Plunkett et al., 2018) from blueberry was used as query to BLAST against the assembled unigene sequences using TBtools (Chen et al., 2020). The BLAST analysis enabled the identification of a unigene (c67315.graph_c0) encoding MYB protein. The full-length open-reading frame of c67315.graph_c0 was amplified from cDNA of Wufanshu berries and confirmed by sequencing. The gene was designated as *VbMYBA* (GenBank Accession No. MW543447) (Supplementary Figure 1). BLASTp search against NCBI non-redundant protein sequences showed that VbMYBA protein have highest matching score to VcMYB1 (AYC35399.1) from *V. corymbosum*. Sequence alignment analysis indicated that VbMYBA protein showed high sequence identity to blueberry VcMYB1 and VcMYBA and contains conserved R2R3 domain and SG6 motif for anthocyanin-promoting MYBs (Figure 3). Nineteen amino acids, three in each R domain and 12 in C-terminal, were different between VbMYBA and VcMYBA (Figure 3). In addition, the N-terminal of VbMYBA protein was 6 aa and 23 aa longer than blueberry VcMYB1 and VcMYBA, respectively (Figure 3). Phylogenetic analysis showed that VbMYBA belongs to SG6 clade, which contains anthocyanin-activating MYB proteins from other plant species and is closely related to the anthocyanin-activators from *Vaccinium* species (Figure 4).

### VbMYBA Is a Strong Anthocyanin Activator

**FIGURE 5:**
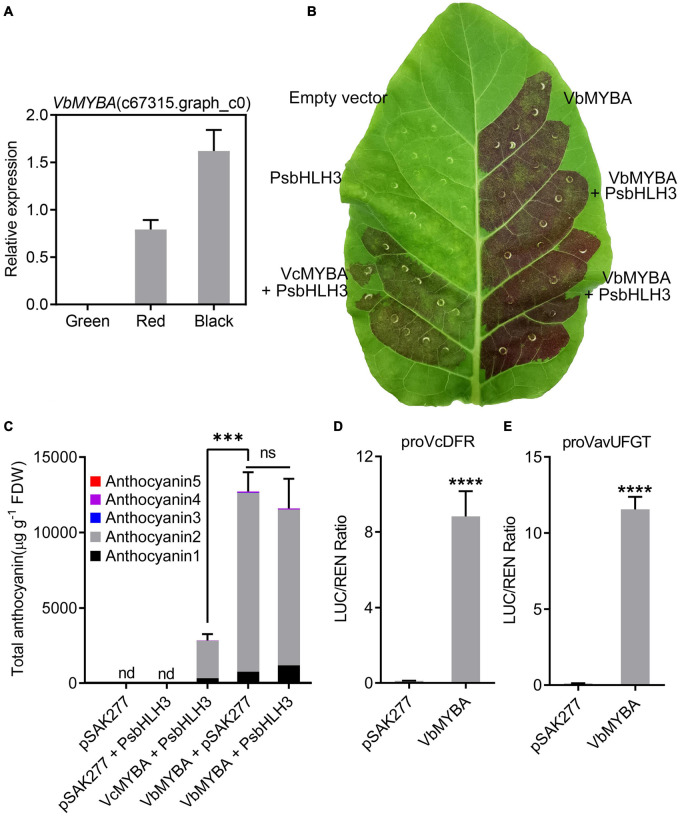
Functional analysis of the *VbMYBA* gene using transient assay in tobacco. **(A)** Relative gene expression of *VbMYBA* in the fruits of Wufanshu. **(B)** Transient expression of *VbMYBA* in tobacco leaf. The photo was taken 5 days after infiltration. Blueberry *VcMYBA*/*PsbHLH3* was used as positive control. **(C)** Anthocyanin content in transformed leaves of tobacco. Anthocyanins were measured as cyanidin-3-galactoside equivalents. Each value represents the mean of three biological replicates. FDW, freeze dry weight. nd, non-detected. Asterisks (***) indicate significant differences among treatments according to *t*-test (*P* < 0.0002). ns, non-significant. Analysis of the interaction of the *VbMYBA* gene with the promoters of VcDFR gene **(D)** from blueberry and *VavUFGT* gene **(E)** from *V. virgatum* using dual-luciferase reporter assay *Nicotiana benthamiana* leaves. Error bars represent the SEs for three replicates. Asterisks (****) indicate significant differences among treatments according to *t*-test (*P* < 0.0001).

Multiple sequences alignment and phylogenetic analysis suggested that VbMYBA was an anthocyanin-activator, similar to VcMYBA. RNA-Seq data along with qRT-PCR analysis indicated that the expression of *VbMYBA* was closely associated with that of anthocyanin biosynthetic genes and anthocyanin accumulation (Figure 5A). The function of VbMYBA was verified using tobacco leaf transient expression assays. Our results indicated that infiltration of *VbMYBA* with or without *PsbHLH3* resulted in strong red pigmentation at infiltration sites 5 days after transformation, but only faint red coloration was found when *VcMYBA* and *PsbHLH3* were co-infiltrated (Figure 5B). Anthocyanin content analysis in leaves of *N. tabacum* indicated that no anthocyanin was detected in the infiltrated areas transformed with empty vector or PsbHLH3 (Figure 5C). A high concentration of anthocyanin was detected in tobacco leaves infiltrated with *VbMYBA*, while co-infiltration of *VbMYBA* and *PsbHLH3* did not result in stronger anthocyanin accumulation in tobacco leaves (Figure 5C). However, the anthocyanin content in tobacco leaves infiltrated with *VcMYBA* and *PsbHLH3* was significantly lower than that in tobacco leaves infiltrated with *VbMYBA* with or without *PsbHLH3* (Figure 5C). Among the anthocyanins detected in tobacco leaves, anthocyanin 1 and 2, which were likely correspond to a cyanidin-3-rutinoside and delphinidin-3-rutinoside, respectively, according to their UV spectra and literature data, were the predominant forms (Figure 5C). In addition, we employed dual-luciferase assays to investigate the function of *VbMYBA*. The promoters of *VcDFR* and *VavUFGT* from blueberry (*V. corymbosum* and *V. virgatum*, respectively) were fused to a luciferase reporter. Infiltration of *VbMYBA* led to strong activation of promoters *VcDFR* and *VavUFGT* (Figures 5D,E). These results demonstrate that VbMYBA is a strong anthocyanin activator and is capable of activating the promoters of *DFR* and *UFGT* from blueberry.

### N-Terminal Amino Acid Residues 1-23 of VbMYBA Is Not Responsible for Its Higher Anthocyanin-Promoting Activity

**FIGURE 6:**
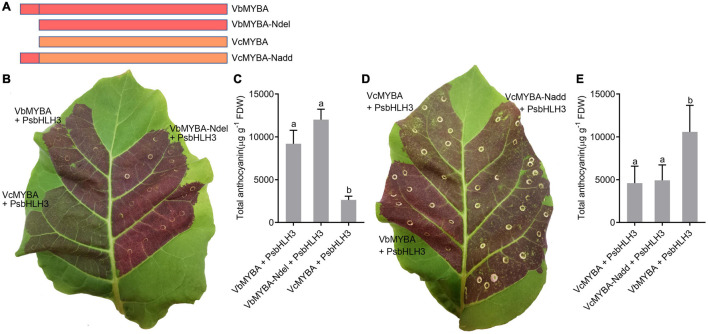
Effect of N-terminal amino acid residues 1-23 of VbMYBA on anthocyanin-promoting activity. **(A)** Schematic diagram of mutants of VbMYBA and VcMYBA. **(B,D)** Transient expression of *VbMYBA*, *VcMYBA* and their mutants in tobacco leaf. The photo was taken 7 days after infiltration. **(C,E)** Anthocyanin content in transformed tobacco leaves. Error bars represent the standard errors for three replicates. Different lowercase letters indicate significant differences among treatments according to one-way ANOVA (*P* < 0.01).

Since the sequence for VbMYBA contained a 23 amino acid-residue insertion in the N-terminal region compared with VcMYBA, we hypothesized that the N-terminal amino acid residues 1-23 of VbMYBA (N^1–23^) are responsible for the capacity for higher anthocyanin-promoting activity. To investigate whether the N^1–23^ is responsible for the divergence of anthocyanin-promoting activity between VbMYBA and VcMYBA, two mutant constructs were generated by deleting the N^1–23^ in VbMYBA (VbMYBA-Ndel) and fusing the N^1–23^ to VcMYBA (VcMYBA-Nadd) (Figure 6A). Anthocyanin-promoting activities of the mutant constructs were tested by tobacco leaf transient expression assays. Our results indicated that deletion of N^1–23^ in VbMYBA or insertion of N^1–23^ to VcMYBA have no significant effects on anthocyanin content in infiltrated tobacco leaves (Figures 6B–E). These results demonstrated that the N-terminal amino acid residues 1-23 of VbMYBA was not responsible for the divergence of anthocyanin-promoting activity between VbMYBA and VcMYBA.

## Discussion

Fruits of *Vaccinium* species are becoming increasingly popular due to their high levels of health-promoting bioactive compounds such as anthocyanins (Krikorian et al., 2010; Norberto et al., 2013; Chu et al., 2017; Shi et al., 2017; Zhou et al., 2020). In this study, the mechanism involved in anthocyanin accumulation in Wufanshu berries was investigated using UHPLC-MS/MS, RNA-Seq, dual-luciferase assays and transient color assays in tobacco leaves.

Anthocyanin composition and content has been reported to be highly variable among *Vaccinium* species and cultivars (Lohachoompol et al., 2008; You et al., 2011; Stevenson and Scalzo, 2012). Our results indicated that ripe Wufanshu berries are rich in a complex set of anthocyanins, including delphinidin, cyanidin, malvidin, peonidin, pelargonidin, and petunidin derivatives. The total anthocyanin content in Wufanshu berries is comparable to the highest content in blueberries as reported by other studies (Stevenson and Scalzo, 2012; Kim et al., 2013; Günther et al., 2020). Pelargonidin and its derivatives were shown to be absent in fruits of *Vaccinium* species such as highbush blueberry (Chung et al., 2016; Chai et al., 2021) and bilberry (Jaakola et al., 2002). However, four derivatives of pelargonidin (pelargonidin-3-*O*-galactoside, pelargonidin-3-*O*-glucoside, pelargonidin-3-*O*-rutinoside and pelargonidin-3,5-*O*-diglucoside) were detected in ripe Wufanshu berries (Supplementary Table 3). In future, it will be interesting to investigate anthocyanin composition of the berries of *V. bracteatum* species and elucidate the mechanisms and pathway enzymes responsible for the accumulation of pelargonidin and its derivatives.

Real-time quantitative RT-PCR analyses showed that whole pathway of anthocyanin biosynthetic genes was upregulated in Wufanshu berries during ripening. *R2R3 MYB* genes have been reported to act as anthocyanin activators in blueberry (Nguyen et al., 2017; Plunkett et al., 2018). Blast analysis enabled us to identify *VbMYBA*, a homolog of the blueberry *R2R3 MYB* gene *VcMYBA* (Plunkett et al., 2018), from the transcriptome of Wufanshu berries. Sequence alignment and phylogenetic analysis suggested that the sub-group 6 VbMYBA is likely to be an anthocyanin activator. qRT-PCR analyses indicated that the expression of *VbMYBA* was well correlated with anthocyanin biosynthetic gene expression and anthocyanin accumulation in Wufanshu berries. Overexpression of *VbMYBA* with or without *PsbHLH3* induced anthocyanin accumulation and red pigmentation in tobacco leaves. Dual-luciferase assays also show that VbMYBA can strongly activate the promoter of anthocyanin biosynthetic genes *VcDFR* and *VavUFGT* from other *Vaccinium* species. These results suggested that VbMYBA is an anthocyanin activator.

The anthocyanin-promoting ability of different *MYB* genes varied significantly (Zhou et al., 2018). Plunkett et al. (2018) show that transient overexpression of blueberry *VcMYBA* with *bHLH3* genes can induce a greater concentration of anthocyanin pigments in tobacco than apple *MdMYB10* and peach *PpMYB10.1*. Zhou et al. (2018) demonstrated that two amino acid changes, Arg/Lys^66^ and Gly/Arg^93^, in the R3 repeat is responsible for anthocyanin-promoting activity divergence between peach *PpMYB10.1* and *PpMYB10.2*. They further demonstrated that reciprocal substitution of Arg/Gly^93^ between PpMYB10.1 and PpMYB10.2 affect their binding affinity to PpbHLH3 and suggested that bHLH-binding affinity is a key factor that determine the anthocyanin-promoting activity of MYB genes (Zhou et al., 2018). However, sequence alignment showed that the two amino acid and bHLH-binding domain were identical between VcMYBA and VbMYBA (Figure 3). The N-terminal region of VbMYBA is longer than VcMYBA, but deletion of 23 amino acid-residue in the N-terminal region of VbMYBA did not reduce its activity. In addition, ligation of the amino acid-residues to the N-terminal of VcMYBA did not enhance its anthocyanin promoting activity. These results suggest that the divergence of anthocyanin-promoting activity between VcMYBA and VbMYBA is likely to be due to the 19 different amino acids that distributed in R domains and C-terminal region. R2 and R3 domains of R2R3-MYBs play pivotal roles in interaction with DNA and determining their DNA binding affinity (Jia et al., 2004; Jiang et al., 2004). It has been reported that mutation of the R2 and R3 domains caused significant alteration in promoter target specificity and DNA-binding affinity of MYB proteins (Williams and Grotewold, 1997; Hernandez et al., 2004; Heppel et al., 2013). The C-terminus contains activation or repression and play an important role in determining the function of MYB proteins (Dubos et al., 2010; Feller et al., 2011). Variation of C-terminus also affect function of MYB proteins (Liu et al., 2016). Further study would be needed to identify the key amino acids responsible for the high anthocyanin-promoting activity of VbMYBA. It is also noteworthy that 23 amino acid-residue in the N-terminal region of VbMYBA should be further verified at a protein level.

It has been demonstrated that bHLHs act as a crucial component in the regulation of anthocyanin biosynthesis. In Arabidopsis, the bHLH factors EGL3 and TT8 are necessary for anthocyanin accumulation in seedlings (Gonzalez et al., 2008). Several studies have indicated that bHLHs are essential for the anthocyanin activating activity of MYB proteins, such as potato R2R3 MYBs (Liu et al., 2016), peach PpMYB10.2 (Zhou et al., 2018), and apple MdMYB10 (Espley et al., 2007) in *Nicotiana tabacum* leaves. Infiltration of some *MYB*s, such as peach *PpMYB10.1* (Zhou et al., 2015) and *PpMYB10.4* (Zhou et al., 2014), Chinese bayberry *MrMYB1* (Niu et al., 2010), litchi *LcMYB1* (Lai et al., 2016), alone activated weak anthocyanin pigmentation in *N. tabacum* leaves, while co-infiltration with *bHLH*s significantly enhanced anthocyanin accumulation. Kiwifruit *AcMYB110* alone was able to induce strong anthocyanin accumulation in *N. tabacum* leaves (Peng et al., 2019). Similarly, infiltration of *VbMYBA* also triggered strong anthocyanin pigmentation in tobacco leaves and co-infiltration with *PsbHLH3* has no significant effects on anthocyanin accumulation (Figures 5A,B and Supplementary Figure 1). One possibility is that *VbMYBA* can recruit bHLHs of *N. tabacum* to activate anthocyanin biosynthetic genes. Intriguingly, its homolog *VcMYBA*, which has a same bHLH-binding domain, cannot activate anthocyanin accumulation in *N. tabacum* leaves when infiltrated alone (Supplementary Figure 2). It will be interesting to investigate the underlying mechanisms that are responsible for MYBs, such as VbMYBA and AcMYB110, to activate anthocyanin accumulation without bHLHs in tobacco leaves.

## Data Availability Statement

The original contributions presented in the study are publicly available. This data can be found here: The raw transcriptome data have been deposited in the NCBI Sequence Read Archive under accession number PRJNA694726.

## Author Contributions

Z-ZF supervised the project. Y-LZ and Z-ZF wrote the manuscript. Y-LZ, Z-ZF, NA, CE, and CA participate in the experiments. KL-W, NA, CE, and RE provided scientific suggestion and revised the manuscript. All authors contributed to the article and approved the submitted version.

## Conflict of Interest

The authors declare that the research was conducted in the absence of any commercial or financial relationships that could be construed as a potential conflict of interest.

## Publisher’s Note

All claims expressed in this article are solely those of the authors and do not necessarily represent those of their affiliated organizations, or those of the publisher, the editors and the reviewers. Any product that may be evaluated in this article, or claim that may be made by its manufacturer, is not guaranteed or endorsed by the publisher.

## Footnotes

1
https://www.ncbi.nlm.nih.gov/orffinder/

2
https://www.genome.jp/tools-bin/clustalw

## Abbreviations

4CL: 4-coumaroyl:CoA-ligase
ANS: anthocyanin synthase
PAL: phenylalanine ammonia-lyase
C4H: cinnamate-4-hydroxylase
CHS: chalcone synthase
COG: Clusters of Orthologous Groups database
DAD: diode array detector
eggNOG: evolutionary genealogy of genes: Non-supervised Orthologous Groups database
ESI: electrospray ion
F3H: flavanone-3-hydroxylase
F3′H: flavonoid3′-hydroxylase
F3′5′H: flavonoid 3′,5′-hydroxylase
FPKM: fragments per kilobase per million mapped reads
DFR: dihydroflavonol-4-reductase
GST: glutathione S-transferase
HPLC: high performance liquid chromatography
KEGG: Kyoto Encyclopedia of Genes and Genomes
KOG: euKaryotic Ortholog Groups of proteins database
MBW: MYB-bHLH-WD40
MS: mass spectrometry
Nr: NCBI non-redundant protein database
Pfam: Pfam-protein family database
qRT-PCR: real-time quantitative RT-PCR
RNA-Seq: RNA-sequencing
Swiss-Prot: Swiss-Prot protein database
UFGT: UDPglucose:flavonoid-3-*O*-glucosyltransferase
GO: Gene Ontology database
UHPLC: ultra-high performance chromatography system.
