# Supplementary figures and images for: Identification of a Strong Anthocyanin Activator, VbMYBA, From Berries of Vaccinium bracteatum Thunb
Source: Front Plant Sci. 2021 Dec 6;12:697212. doi: 10.3389/fpls.2021.697212 (PMC8685453; doi:10.3389/fpls.2021.697212)

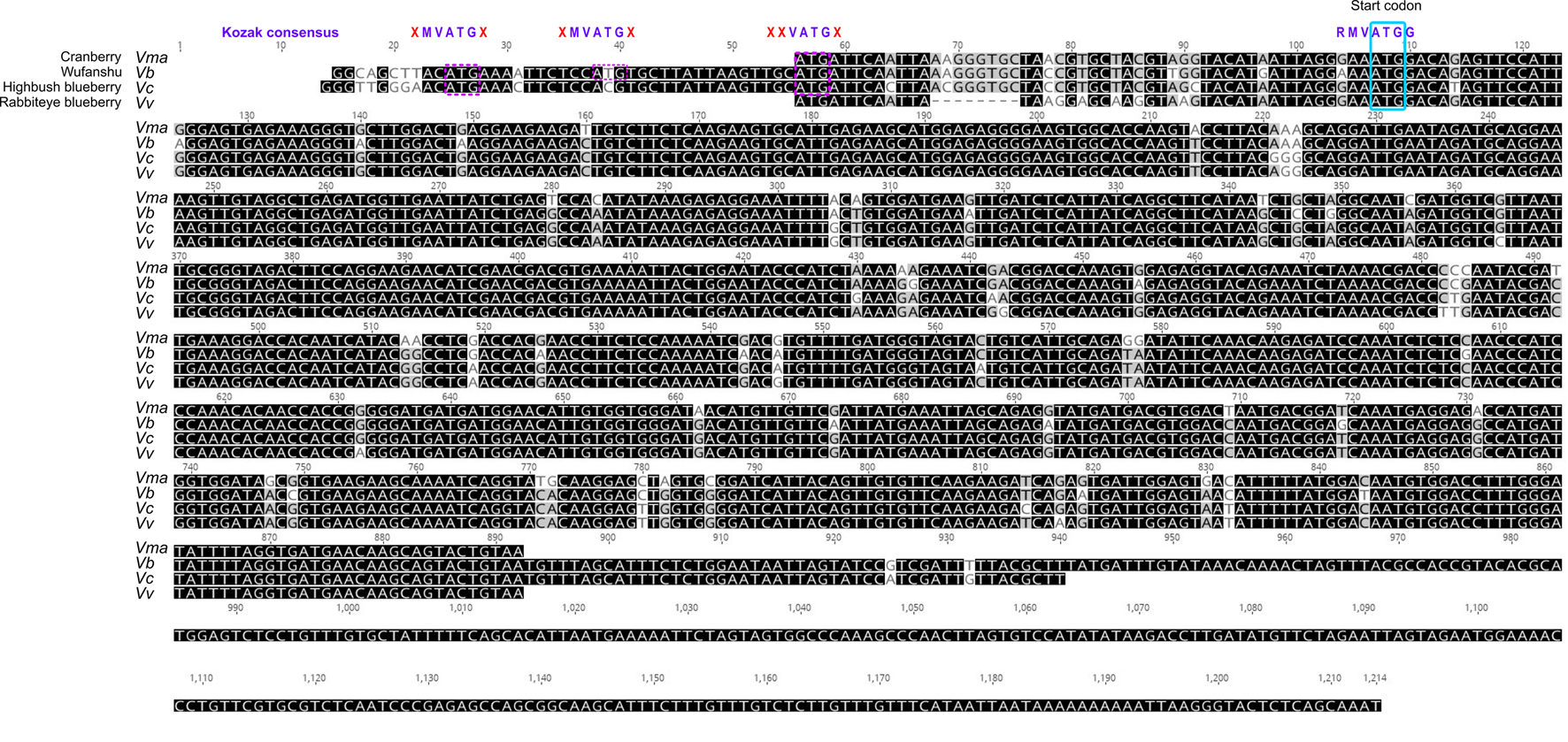

Supplement: Supplementary file 1 [file Image_1.TIF]

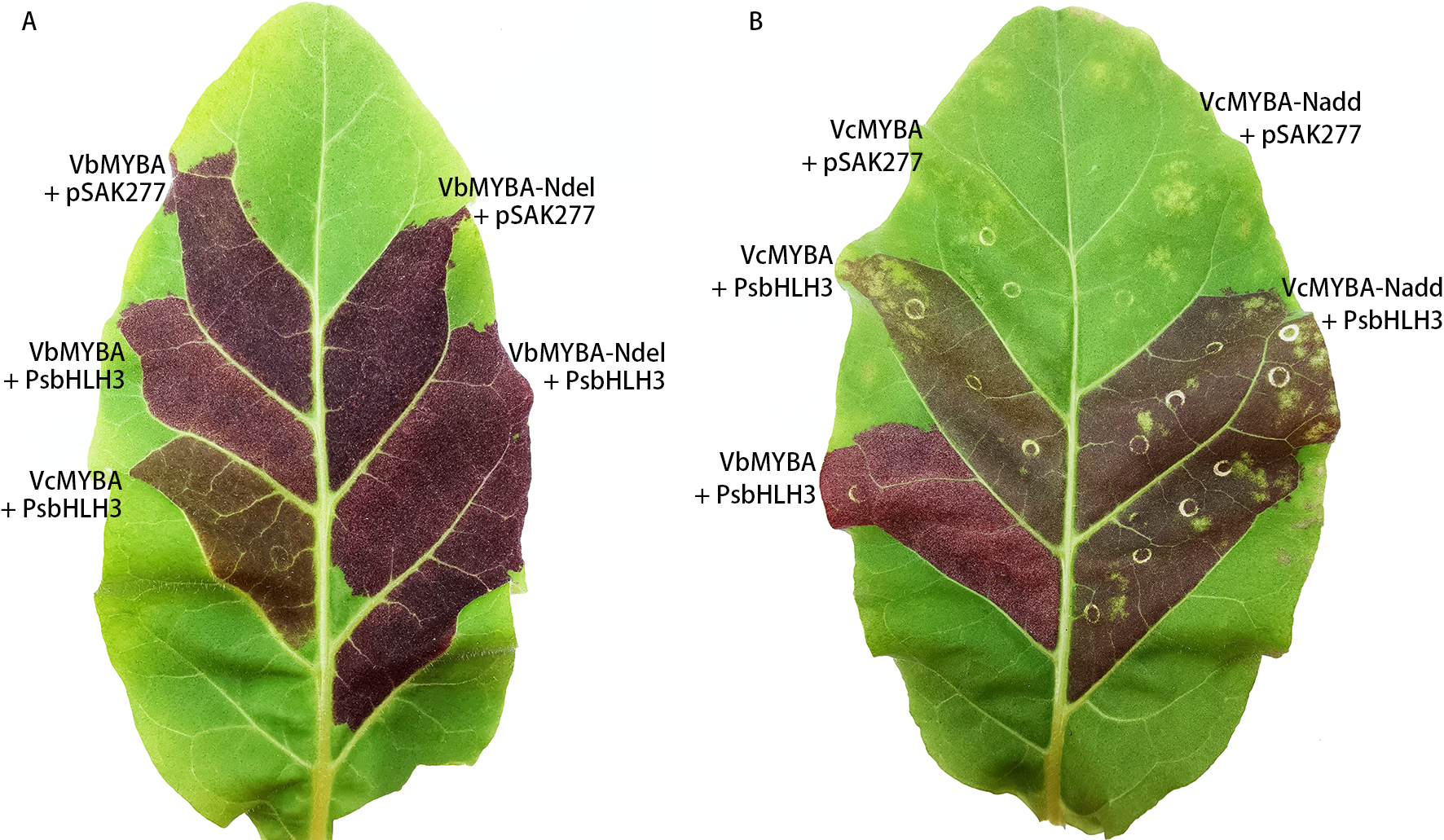

Supplement: Supplementary file 2 [file Image_2.TIF]
