# Supplementary material for: Identification of a Strong Anthocyanin Activator, VbMYBA, From Berries of Vaccinium bracteatum Thunb
Source: Front Plant Sci. 2021 Dec 6;12:697212. doi: 10.3389/fpls.2021.697212 (PMC8685453; doi:10.3389/fpls.2021.697212)
Supplement: Supplementary file 3 [file Table_1.DOCX]

| **Table S1 Primer sequences for qRT-PCR analysis** | | |
| --- | --- | --- |
| Genes | Forward primer | Reverse primer |
| *VbPAL* | ACGTCGCCCGGTGAGGAATG | AATTGGCAGAGGGGCGCCAT |
| *Vb4CL* | GACGGCGAGAATCCGCACCT | GCCGCCCCGACTCTCAAACT |
| *VbC4H* | CAGACGAAGCTCCGGCACGA | ATCCGGAGGCGGAGGGTCTC |
| *VbCHS* | ATTGGGCAAAGAGGCTGCTGTG | CATGTCGACGCCGGAGGTGG |
| *VbF3H* | CACGGCGTGGATTCGAGTCTG | GGGCCCGGATCGGGTATGAG |
| *VbF3H* | ACCCGGCTCCCAATGCAACT | GCCTCCAATTGGGCTTCTGCG |
| *VbF3’H* | GCCGCTGATGCACCTCTGGA | TCCTAAGCATCCGCCACCGC |
| *VbF3’ 5’H* | GCCATGGTCGAATCAAGCCAAC | GGACAAACACGCGACGCTTGAG |
| *VbDFR* | CGCCGGGTTCATCGGCTCA | CCGCCTTCCACAGCGTTAGGT |
| *VbUFGT* | GGGGTGCCATGGATCACGCT | CGTTTTCGCGTCCCGCAGTG |
| *VbGST* | AGGGCAGCATGCCCACAGAG | TCCAAAAGGCTGGCGGAGCA |
| *VbMYBA* | ACAACCACCGGGGGATGATG | GGCCTCCTCATTTGCTCCGT |
| *ACTIN* | ATGAAGCGCAGTCCAAGCGT | GGGTGCTCCTCTGGGGCTAC |
