# Supplementary material for: Identification of a Strong Anthocyanin Activator, VbMYBA, From Berries of Vaccinium bracteatum Thunb
Source: Front Plant Sci. 2021 Dec 6;12:697212. doi: 10.3389/fpls.2021.697212 (PMC8685453; doi:10.3389/fpls.2021.697212)
Supplement: Supplementary file 4 [file Table_2.DOCX]

**Table S2 Sequences of primers used for vector construction**

| Primer name | Sequence | Description |
| --- | --- | --- |
| VbMYBAOEF | actagtggatccaaagaattcATGTGCTTATTAAGTTGCATGATTCA | Primers used for inserting *VbMYBA* into vector pSAK277 |
| VbMYBAOER | agaagtactctcgagaagcttTTACAGTACTGCTTGTTCATCACCTAA |  |
| VcMYBAOEF | actagtggatccaaagaattcATGGACATAGTTCCATTGGGAG | Primers used for inserting *VcMYBA* into vector pSAK277 |
| VcMYBAOER | agaagtactctcgagaagcttTTACAGTACTGCTTGTTCATCACCT |  |
| VbMYB-NdelOEF | actagtggatccaaagaattcATGGACAGAGTTCCATTAGGAGTG | Primers used for inserting fragment (70-855bp) of *VbMYBA* Vector of PsMYB10.2 |
| VbMYB-NdelOER | agaagtactctcgagaagcttTTACAGTACTGCTTGTTCATCACCTAA |  |
| VbMYBNF | actagtggatccaaagaattcATGTGCTTATTAAGTTGCATGATTCA | Primers used for amplifying N-treminal of *VbMYBA* (1-69bp) |
| VbMYBNR | CTCCCAATGGAACTATGTCCATTTTCCTAATCATGTACCAACGTAG |  |
| VcMYBF | CTACGTTGGTACATGATTAGGAAAATGGACATAGTTCCATTGGGAG | Primers used for amplifying *VcMYBA* contains overlap with N-treminal of *VbMYBA*(1-69bp) |
| VcMYBR | agaagtactctcgagaagcttTTACAGTACTGCTTGTTCATCACCT |  |
| VbMYB-NaddOEF | actagtggatccaaagaattcATGTGCTTATTAAGTTGCATGATTCA | Primers used for inserting *VcMYBA-Nadd* into vector pSAK277 |
| VbMYB-NaddOER | agaagtactctcgagaagcttTTACAGTACTGCTTGTTCATCACCT |  |
