# Supplementary material for: Identification of a Strong Anthocyanin Activator, VbMYBA, From Berries of Vaccinium bracteatum Thunb
Source: Front Plant Sci. 2021 Dec 6;12:697212. doi: 10.3389/fpls.2021.697212 (PMC8685453; doi:10.3389/fpls.2021.697212)
Supplement: Supplementary file 6 [file Table_4.DOCX]

**Table S4 Summary of sequencing and mapping results**

| Sample Name | Clean Reads | Clean bases (bp) | GC Content | %≥Q30 | Map Rate |
| --- | --- | --- | --- | --- | --- |
| Green | 21,844,347 | 6,522,355,904 | 46.36% | 87.67% | 63.25% |
| Black | 23,478,300 | 6,968,921,256 | 46.78% | 88.14% | 62.50% |
