# Supplementary material for: Identification of a Strong Anthocyanin Activator, VbMYBA, From Berries of Vaccinium bracteatum Thunb
Source: Front Plant Sci. 2021 Dec 6;12:697212. doi: 10.3389/fpls.2021.697212 (PMC8685453; doi:10.3389/fpls.2021.697212)
Supplement: Supplementary file 7 [file Table_5.DOCX]

**Table S5 Statistics of unigenes**

| Length Range | Transcript | Unigene |
| --- | --- | --- |
| 200-300 | 36,299(27.47%) | 33,066(37.66%) |
| 300-500 | 28,746(21.75%) | 21,011(23.93%) |
| 500-1000 | 31,623(23.93%) | 17,415(19.83%) |
| 1000-2000 | 23,973(18.14%) | 11,122(12.67%) |
| 2000+ | 11,501(8.70%) | 5,197(5.92%) |
| Total Number | 132,142 | 87,811 |
| Total Length | 109,951,836 | 59,048,744 |
| N50 Length | 1,328 | 1,063 |
| Mean Length | 832.07 | 672.45 |
