# Supplementary material for: Identification of a Strong Anthocyanin Activator, VbMYBA, From Berries of Vaccinium bracteatum Thunb
Source: Front Plant Sci. 2021 Dec 6;12:697212. doi: 10.3389/fpls.2021.697212 (PMC8685453; doi:10.3389/fpls.2021.697212)
Supplement: Supplementary file 8 [file Table_6.DOCX]

**Table S6 Summary of functional annotations for the assembled unigenes**

| Database | Number of annotated unigenes | Percentage of annotated unigenes (%) | 300 ≤ Length <1000 | Length ≥1000 bp |
| --- | --- | --- | --- | --- |
| Nr | 41204 | 46.92 | 14824 | 13457 |
| Swiss-Prot | 22792 | 25.96 | 8597 | 9498 |
| GO | 24120 | 27.47 | 8498 | 8057 |
| COG | 13991 | 15.93 | 4431 | 5253 |
| KOG | 22664 | 25.81 | 7954 | 8434 |
| KEGG | 14952 | 17.03 | 5519 | 5276 |
| Pfam | 26834 | 30.56 | 9204 | 11340 |
| eggNOG | 38879 | 44.28 | 14037 | 12236 |
| All | 43355 | 49.37 | 15599 | 13553 |
